# Relationship between foot function and medial knee joint loading in people with medial compartment knee osteoarthritis

**DOI:** 10.1186/1757-1146-6-33

**Published:** 2013-08-08

**Authors:** Pazit Levinger, Hylton B Menz, Adam D Morrow, John R Bartlett, Julian A Feller, Neil R Bergman

**Affiliations:** 1 Institute of Sport, Exercise and Active Living, College of Sport and Exercise Science, Victoria University, Melbourne Vic 8001, Australia; 2 Lower Extremity and Gait Studies Program, Faculty of Health Sciences, La Trobe University, Melbourne Vic 3086, Australia; 3 Warringal Medical Centre, Melbourne Vic 3084, Australia

**Keywords:** Knee osteoarthritis, Foot motion, Knee adduction moment

## Abstract

**Background:**

Dynamic joint loading, particularly the external knee adduction moment (KAM), is an important surrogate measure for the medio-lateral distribution of force across the knee joint in people with knee osteoarthritis (OA). Foot motion may alter the load on the medial tibiofemoral joint and hence affect the KAM. Therefore, this study aimed to investigate the relationship between tibia, rearfoot and forefoot motion in the frontal and transverse planes and the KAM in people with medial compartment knee OA.

**Method:**

Motion of the knee, tibia, rearfoot and forefoot and knee moments were evaluated in 32 patients with clinically and radiographically-confirmed OA, predominantly in the medial compartment. Pearson’s correlation coefficient was used to investigate the association between peak values of tibia, rearfoot and forefoot motion in the frontal and transverse planes and 1^st^ peak KAM, 2^nd^ peak KAM, and the knee adduction angular impulse (KAAI).

**Results:**

Lateral tilt of the tibia was significantly associated with increased 1^st^ peak KAM (*r* = 0.60, *p* < 0.001), 2^nd^ peak KAM (*r* = 0.67, *p* = 0.001) and KAAI (*r* = 0.82, *p* = 0.001). Increased peak rearfoot eversion was significantly correlated with decreased 2^nd^ peak KAM (*r* = 0.59, *p* < 0.001) and KAAI (*r* = 0.50, *p* = 0.004). Decreased rearfoot internal rotation was significantly associated with increased 2^nd^ peak KAM (*r* = −0.44, *p* = 0.01) and KAAI (*r* = −0.38, *p* = 0.02), while decreased rearfoot internal rotation relative to the tibia was significantly associated with increased 2^nd^ peak KAM (*r* = 0.43, *p* = 0.01). Significant negative correlations were found between peak forefoot eversion relative to the rearfoot and 2^nd^ peak KAM (*r* = −0.53, *p* = 0.002) and KAAI (*r* = −0.51, *p* = 0.003) and between peak forefoot inversion and 2^nd^ peak KAM (*r* = −0.54, *p* = 0.001) and KAAI (*r* = −0.48, *p* = 0.005).

**Conclusion:**

Increased rearfoot eversion, rearfoot internal rotation and forefoot inversion are associated with reduced knee adduction moments during the stance phase of gait, suggesting that medial knee joint loading is reduced in people with OA who walk with greater foot pronation. These findings have implications for the design of load-modifying interventions in people with knee OA.

## Background

Knee osteoarthritis (OA) is a chronic debilitating condition, affecting a substantial number of older people worldwide [1,2]. People with knee OA suffer from pain and difficulties in performing activities of daily living. OA in the medial compartment of the knee is highly prevalent and has been attributed to the increased load transmitted across the medial compartment of the knee joint [3]. Although several factors have been associated with the incidence and progression of OA, particularly medial compartment knee OA, the aetiology of knee OA is not fully understood. Biomechanical factors associated with joint loading have been the focus of recent studies as an important element in the pathogenesis of knee OA.

Dynamic joint loading, particularly the external knee adduction moment (KAM), has received attention as an important surrogate measure of the medio-lateral distribution of force across the knee joint. Although the evidence for the contribution of KAM to the development of knee OA is inconsistent [4], several studies have shown increased KAM to be associated with knee OA severity and varus malalignment [5]. Consequently, several treatment strategies, including load modifying interventions, have been suggested to reduce the load on the medial compartment of the knee by altering the KAM [6-12].

The KAM is influenced by variation in lower limb alignment and motion during gait [13-17]. Varus limb alignment, which is commonly observed in people with medial compartment knee OA, has been shown to increase the incidence and progression of knee OA [18-20]. Recent studies have also reported that people with medial compartment knee OA have a relatively pronated foot posture [21-23] and demonstrate foot kinematic patterns that are indicative of a less mobile, everted foot type [24] compared to controls. Moreover, the degree of varus alignment may also affect foot motion during walking which may lead to a compensatory response to allow typical function of the foot during ambulation [24]. Footwear and orthotic interventions, therefore, have been studied as a method for altering medial knee loading by altering foot motion [6-12].

The mechanism by which footwear and orthotic interventions aim to reduce the knee adduction moment is by pronating the foot through lateral inclination of the insole (thereby laterally shifting the centre of pressure) [25]. However, it is unclear if variation in foot motion, particularly foot eversion, influences the KAM. In order to better understand how knee joint loading is influenced by lower limb motion, this study investigated the relationship between tibia, rearfoot and forefoot motion in the frontal and transverse planes and KAM in people with medial compartment knee OA. We hypothesised that kinematic parameters indicative of greater foot pronation (internal tibial rotation, frontal plane rearfoot eversion and frontal plane forefoot inversion) would be associated with a reduction in medial knee joint loading.

## Methods

This project was part of a larger study that investigated gait (swing phase mechanics, particularly minimum foot clearance), balance and falls risk in people before and after knee arthroplasty. A power calculation to determine the sample size, therefore, was based on minimum foot clearance parameters. Data from a previous study [26] which investigated the toe clearance of elderly fallers and non-fallers were used to determine the number of participants required. A sample size calculation indicated that for 80% power and a *p* value of 0.05 at least 25 participants were required. To mitigate the possible effect of subject drop out for the surgical group, a total of 32 participants were considered to be sufficient. Thirty two participants (16 females, average age 65.8 ± 7.5 yr, height 168.8 ± 9.5 and body weight 85.1 ± 13.6kg) with diagnosed OA predominantly in the medial compartment of the knee, determined by radiographic assessment [24], participated in the study. Detail of the foot posture of the participants has previously been reported [21]. The severity of knee OA was based on the loss of joint space determined by an orthopaedic surgeon from radiographic images [27] and was graded as follows: 1- less than a half of joint space loss (mild), 2 - more than a half of joint space loss; bone on bone (moderate) and 3 - bone deformity/loss of bone (severe). Each compartment of the knee joint (medial compartment, lateral compartment and patellofemoral compartment) was graded and participants with predominantly medial compartment knee OA (severity grade 2–3) were included in the study. Sixteen participants had moderate severity of OA (grade 2) and 16 participants had severe OA (grade 3) based on radiographic assessment [27]. Participants were included if they were able to walk independently and were excluded if they had uncontrolled systemic disease and or a pre-existing neurological or other orthopaedic condition that affected their walking. Participants were recruited from the La Trobe University Medical Centre, the Warringal Private Medical Centre and through advertisements in local newspapers. Ethics approval was obtained from the Faculty of Health Sciences Human Ethics Committee, La Trobe University. All participants were informed about the nature of the study and signed a consent form prior to participation.

### Procedure

#### Instrumentation

A three dimensional motion analysis system (Vicon MX, Vicon Motion System Ltd, Oxford, England) with 10 cameras (8 MX3 and 2 MX40) was used to capture and analyse motion of the lower leg with a sampling frequency of 100Hz. Two force plates (Kistler, type 9865B, Winterthur, Switzerland and AMTI, Watertown, MA, USA) (1000Hz) were used to capture ground reaction forces and identify gait cycle events. The marker trajectories and force platform data were captured synchronously using the Vicon Nexus software package. The force plate data were then re-sampled at 100Hz for the calculation of knee joint moments.

#### Kinematic evaluation

Participants were required to attend a single testing session at the gait laboratory at La Trobe University. Lower leg and foot motion of the symptomatic leg (or the most symptomatic leg in a case of bilateral involvement) was assessed. To assess the three dimensional motion of the lower limb including, knee, tibia, rearfoot and forefoot and knee moments in the frontal plane, retro-reflective markers were attached on anatomical landmarks over the lower legs in accordance with the Oxford Foot Model (OFM) marker set and Plug In Gait (PIG) [28] as described by Stebbins *et al*. [29]. Retro-reflective markers were then placed over the anatomical land marks on the pelvis, thigh, tibia, rearfoot and forefoot as described in details in Levinger *et al*. [24]. The OFM modelled the tibia, rearfoot and forefoot as rigid segments. The tibial segment was comprised of markers placed on the medial malleolus, the lateral malleolus, the anterior aspect of the tibial crest, the tibial tuberosity and the head of the fibula. The rearfoot segment was defined by placing markers on the sustentaculum tali, the lateral calcaneus, the heel (distal part of the calcaneus), the posterior proximal calcaneus and a peg marker was placed on the posterior calcaneus between the heel and proximal calcaneus markers. The forefoot segment was defined by placing markers on the most distal, medial aspect of the first metatarsal shaft, the most proximal and distal lateral aspects of the fifth metatarsal shaft, and midway between the second and third metatarsal heads.

Prior to kinematic evaluation of the lower leg motion, a relaxed standing calibration trial was captured with knee alignment devices (KAD, Motion Lab Systems Inc. LA, USA). Several markers, used only in the static trials (medial malleoli, proximal heel, and first metatarsal), were removed prior to the dynamic trials as described in Stebbins *et al*. [29]. The locations of the joints centre were calculated from PIG [28]. Moreover, the location of the knee joint centre, calculated from PIG, was further used in the OFM for the tibia segment definition.

Participants were asked to walk at a comfortable walking pace along a 12m walkway and five successful trials were collected for each leg. A successful trial was defined when the participant’s foot landed on the centre of the force plate without any interference to their gait. For each trial, gait events were detected using vertical ground reaction force data to determine initial foot contact and toe off. Multiple trials were practiced until participants were comfortable and walking with consistent velocity. The peak values of interest (maximum value during the stance phase) of each trial were extracted separately; the average of the five trials was then used in the analysis. All gait variables of interest generated by the model were normalised to the gait cycle and timing of peak angular variables were then expressed as a percentage of the gait cycle.

**Figure 1:**
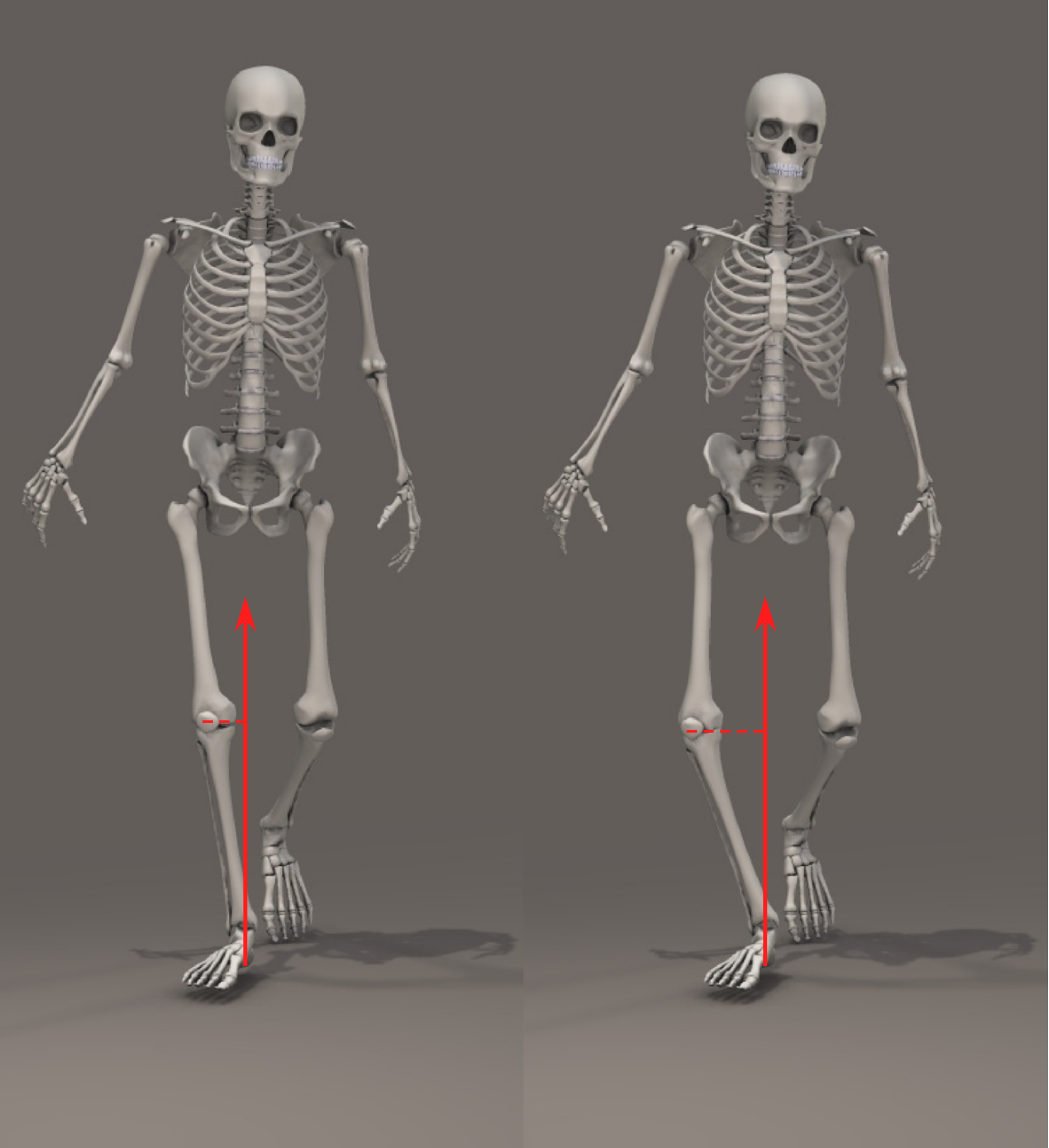
The knee adduction moment (KAM) increases when walking with greater varus alignment of the knee (shown on the right) as the perpendicular distance of the ground reaction force vector from the knee joint centre is greater, resulting in a longer moment arm.

**Figure 2:**
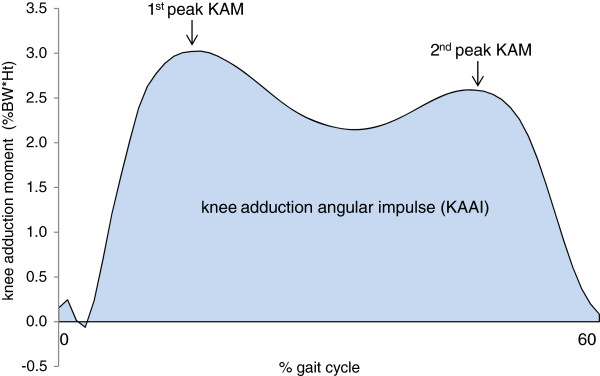
**Frontal plane external KAMs: 1**^**st **^**peak KAM, 2**^**nd **^**peak KAM and knee adduction angular impulse (KAAI), which represents the area under the curve.**

The magnitude of peak angular motion of the tibia, rearfoot relative to the global coordinate system (laboratory), rearfoot relative to the tibia and forefoot relative to the rearfoot in the frontal and transverse planes during the gait cycle were extracted including the following angles: (i) tibia lateral tilt and internal/external rotations (ii) peak rearfoot eversion/inversion and internal/external rotation relative to the tibia; (iii) rearfoot eversion/inversion and internal/external rotation relative to the global reference system (laboratory); (iv) peak forefoot abduction/adduction; and eversion/inversion. Knee frontal plane angular motion (knee varus) during initial contact and during stance (peak knee varus) and external KAM (normalised to % of body weight*height) including 1^st^ peak and 2^nd^ peak were also extracted. Knee adduction angular impulse (KAAI - the integral of the frontal plane knee moment over the stance phase of the gait cycle) [30] was also calculated. Figure 1 depicts the influence of knee alignment on the KAM, and Figure 2 depicts the three kinetic variables extracted from this data (1^st^ peak KAM, 2^nd^ peak KAM and KAAI).

#### Knee pain, function and stiffness

Clinical severity of OA including physical function, pain and stiffness were assessed using the Western Ontario and McMaster University Osteoarthritis Index (WOMAC) [31]. This index, using 10mm visual analogue scale, assesses the severity of the knee pain during 5 daily activities (range 0 – 500), stiffness (range 0 – 200), and the severity of impairment of lower-extremity function during 17 activities (0 – 1700). A score of zero represents no pain or difficulty with physical function and higher scores represent worse functional health. All three subcategories are summed to give a global WOMAC score (range 0 – 2400).

#### Statistical analysis

Pearson’s correlation coefficient was used to investigate the relationship between peak KAMs (1^st^ peak KAM and 2^nd^ peak KAM), KAAI and the following parameters: peak values of tibia, rearfoot (both relative to the laboratory and relative to the tibia) and forefoot motion in the frontal and transverse planes.

## Results

**Figure 3:**
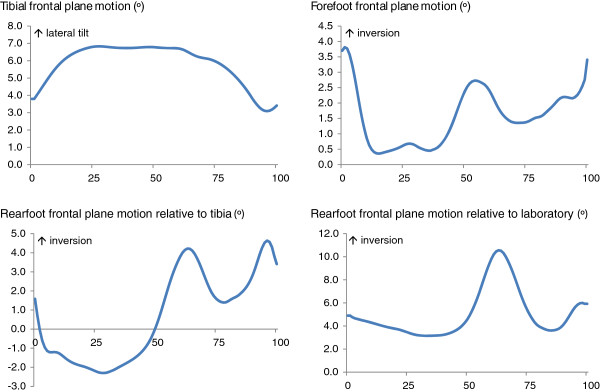
Mean motion of the tibia, rearfoot and forefoot in the frontal plane expressed relative to the percentage of the gait cycle.

**Figure 4:**
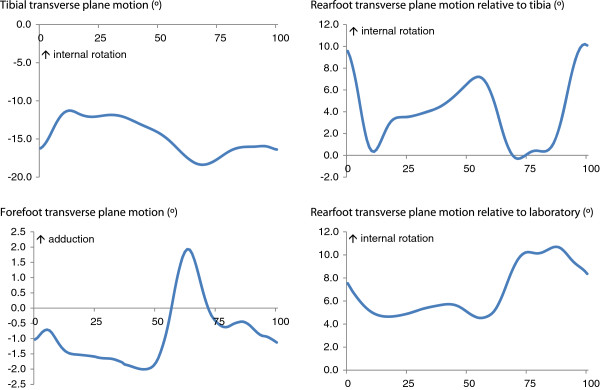
Mean motion of the tibia, rearfoot and forefoot in the transverse plane expressed relative to the percentage of the gait cycle.

Mean ± standard deviation external KAMs (% bodyweight * height) were as follows: 1^st^ peak KAM = 3.3 ± 1.6, 2^nd^ peak KAM = 2.8 ± 1.1 and KAAI = 1.26 ± 0.5. Correlations between foot motion and KAM-related variables are shown in Table 1. Angular motion of the tibia, rearfoot and forefoot in the frontal and transverse planes are presented in Figures 3 and 4. The knee OA group reported mild pain of 171.8 ± 99.9, function 502.5 ± 330.9 and stiffness 83.7 ± 49.8 with WOMAC total score of 758.1 ± 447.1.

**Table 1:** Correlations between peak rearfoot and forefoot motion in the frontal plane and peak knee adduction moments (KAM) and knee adduction angular impulse (KAAI)

	**1**^**st **^**peak KAM**		**2**^**nd **^**peak KAM**		**KAAI**	
**Parameters**	***r *value**	***p *value**	***r *value**	***p *value**	***r *value**	***p *value**
Tibia relative to laboratory						
Lateral tilt	0.60*	< 0.001	0.67*	0.001	0.82*	< 0.001
Internal Rotation	0.27	0.13	0.17	0.34	0.19	0.28
External Rotation	0.14	0.44	0.19	0.29	0.14	0.43
Rearfoot relative to the tibia						
Peak eversion	−0.12	0.48	0.25	0.16	0.07	0.69
Peak inversion	−0.08	0.63	0.10	0.56	0.03	0.85
Internal Rotation	−0.02	0.89	0.43*	0.01	0.29	0.10
External Rotation	−0.22	0.20	0.28	0.10	0.07	0.67
Rearfoot relative to laboratory						
Peak eversion	0.21	0.24	0.59*	< 0.001	0.50*	0.004
Peak inversion	0.07	0.69	0.33	0.06	0.33	0.05
Internal Rotation	−0.18	0.30	−0.44*	0.01	−0.38*	0.02
External Rotation	−0.22	0.20	−0.54*	0.001	−0.48*	0.005
Forefoot relative to rearfoot						
Peak eversion	−0.26	0.13	−0.53*	0.002	−0.51*	0.003
Peak inversion	−0.28	0.11	−0.54*	0.001	−0.48*	0.005
Abduction	0.18	0.30	0.14	0.42	0.13	0.47
Adduction	0.19	0.28	0.13	0.47	0.14	0.42

Note: A positive correlation indicates increased peak rearfoot eversion and decreased rearfoot inversion is associated with decreased adduction moments. A negative correlation indicates reduced rearfoot internal rotation and increased external rotation relative to the laboratory is associated with greater 2^nd^ peak KAM and KAAI.

* significant at *p* < 0.05.

Greater lateral tilt of the tibia was significantly correlated with increased KAMs and KAAI (*r* = 0.60 to 0.82, *p* = 0.001). No correlations were found between tibial rotation and KAMs (Table 1). Significant positive correlations were found between peak rearfoot eversion relative to the laboratory and 2^nd^ peak KAM (*r* = 0.59, *p* < 0.001) and KAAI (*r* = 0.50, *p* = 0.004), indicating decreased adduction moments with greater rearfoot eversion. Reduced rearfoot internal rotation relative to the laboratory was significantly correlated with greater 2^nd^ peak KAM (*r* = −0.44, *p* = 0.01) and KAAI (*r* = −0.38, *p* = 0.02). Similarly, increased rearfoot external rotation relative to the laboratory was significantly correlated with greater 2^nd^ peak KAM (*r* = −0.54, *p* = 0.001) and KAAI (*r* = −0.48, *p* = 0.005). No significant correlations were found between either peak rearfoot eversion or inversion relative to the tibia and any of the KAMs. Reduced rearfoot internal rotation relative to the tibia was significantly correlated with increased 2^nd^ peak KAM (*r* = 0.43, *p* = 0.01).

Significant negative correlations were found between peak forefoot eversion and 2^nd^ peak KAM (*r −*0.53, *p* = 0.002) and between peak forefoot inversion and 2^nd^ peak KAM (*r* = −0.54, *p* = 0.001). Similar correlations were also found between forefoot peak eversion and inversion and KAAI. The forefoot is generally inverted during the stance phase as it is affected by rearfoot eversion [24]. Therefore, a negative correlation indicates an association between *increased* forefoot inversion and *reduced* knee impulse and KAMs.

## Discussion

Load-modifying interventions have been proposed as a strategy to reduce medial compartment knee loading (by reducing the external KAM) in people with knee OA, however equivocal findings have been reported regarding their effectiveness [10,25,32,33]. These strategies rely on altering knee joint loading by modifying movement of the foot, suggesting that by influencing foot motion, the moment arm of the ground reaction force that passes medially to the knee joint centre is reduced (Figure 1). Understanding the relationship between lower leg and foot motion and KAMs can therefore provide useful information to help optimise intervention strategies. In the present study, we found several associations between foot and tibia motion and external adduction moments at the knee which may influence the design of load-altering interventions for knee OA.

Knee varus is frequently observed in people with medial compartment knee OA, with evidence suggesting that knee varus alignment increases the incidence and progression of OA [18-20]. We found significant correlations between lateral tibial tilt and KAMs and KAAI, indicating that greater tibial tilt increases the load on the medial compartment of the knee joint. These findings were expected, as greater tibial lateral tilt would increase the perpendicular distance of the ground reaction force vector from the knee joint centre, resulting in a greater moment arm and adduction moment (Figure 1). No correlation, however, was found in the transverse plane. Interestingly, an association between OA progression and torsional deformity in the tibia has previously been reported, as a decrease in tibial external rotation was accompanied by an increase in disease severity [34]. We have previously reported that people with knee OA have greater tibial internal rotation compared to aged-matched controls [35], and a reduction in tibial internal rotation was observed in patients who underwent realignment of the knee following knee replacement surgery [35]. Furthermore, internal torsion and varus deformity have been associated with increased loads on the medial compartment of the knee [36]. The lack of correlation in the present study between tibia rotation and KAMs may be related to a possible restriction in tibial motion as patients may be “pushed” to the end range of motion due to years of walking in the same pattern. However, due to the cross-sectional design of these studies, we are unable to infer the direction of causation between tibial rotation and the load on the medial compartment of the knee.

A significant association between rearfoot eversion relative to the laboratory and KAMs and KAAI was found, indicating that increased foot pronation is associated with reduced medial knee joint loading. In our previous work, the same knee OA group exhibited a relatively pronated foot type compared to an age-matched control group [21]. Greater internal rotation and reduced external rotation of the rearfoot relative to the lab were also associated with reduced 2^nd^ peak KAM and KAAI. Movement of the rearfoot in the frontal plane is coupled to internal rotation of the rearfoot, as previously reported in flat arched-feet [37]. Consequently, greater rearfoot eversion and internal rotation were associated with reduction of the overall medial loading during the stance phase of gait. Unlike rearfoot motion relative to the tibia, rearfoot motion relative to the laboratory is an independent measure of absolute rearfoot motion which may therefore more closely represent the link between rearfoot motion and knee moments.

Significant correlations were also found between forefoot frontal plane motion and peak KAMs, indicating that increased forefoot inversion was related to reduced knee impulse and KAM during mid and late stance phase. Due to the coupling movement between the rearfoot and forefoot [38], inversion of the forefoot during stance phase is affected by the degree of rearfoot eversion, therefore the greater rearfoot eversion in the knee OA group would make the forefoot relatively inverted. It is also possible that motion of the midfoot in the frontal plane affects medial knee loading, however due to the absence of a midfoot segment in the Oxford foot model, we are unable to determine the contribution of midfoot motion to altered medial knee loading. Kinematic foot models which allow for more detailed analysis of the midfoot may be of additional benefit to better understand foot function in people with medial compartment knee OA. Nevertheless, based on our current findings, the correlations found between frontal plane rearfoot and forefoot motion and peak KAMs suggest that those with greater peak rearfoot eversion and forefoot inversion exhibited reduced medial knee joint loading during the mid- to late stance phase of gait.

Interestingly, the associations found between foot kinematics and KAMs did not involve the 1^st^ peak KAM, which is most often targeted with load-altering interventions. There are a number of possible explanations for this. Firstly, there are differences in timing of when these peaks occur during gait. The 1^st^ peak KAM for the knee OA group occurred at early stance (average 19.1% gait cycle) while peak rearfoot eversion occurred at 30% of the gait cycle. Secondly, it is possible that the movement of the midfoot may be related to medial knee loading, as midfoot motion can compensate for rearfoot motion. Further investigation, however, is required to ascertain this. Lastly, previous studies have suggested that there may be distinct subgroups of individuals based on timing patterns of rearfoot frontal plane motion [39]. Participants with an “early” rearfoot eversion pattern (ie. rapid eversion in the first 10% gait cycle) may be more likely to exhibit changes in KAM associated with load-altering interventions, which may explain why these interventions are more effective in some subpopulations than others. It is also important to acknowledge that KAAI has been shown to be a more sensitive mechanical joint loading parameter than peak KAMs [40]. Given that the KAAI takes into account both the magnitude and duration of knee medial loading, the correlation between greater peak rearfoot eversion and forefoot inversion and reduced KAAI may indicate overall reduction of medial knee joint loading during stance.

Reports of the biomechanical effects of different load modifying interventions (orthotics and shoes) have been inconsistent [10,25,32,33]. While some studies investigating lateral wedged insoles have reported a reduction in the KAMs [10,11,33,41], others have reported an *increase* in KAM [25,32,33]. Different insole lengths have also shown different responses, with full length insoles being more effective at reducing KAM than heel wedges [10]. These findings support the suggestion that there may be sub-groups that better respond to lateral wedged insoles, and that variability in response to orthotic intervention may be evident in people with medial compartment knee OA [24]. Footwear-related interventions, such as variable stiffness shoes, have also demonstrated a reduction in the KAM [6,12,42] with evidence suggesting a reduction in the medial compartment *in vivo* contact force [12]. It may be possible that insole interventions which aim at modifying motion of the whole foot (rearfoot, midfoot and forefoot), such as shoes and full length insoles, may be more effective due to their effect on forefoot frontal plane motion in addition to altering rearfoot motion. However, due to the potential high variability in response to load modifying interventions, appropriate individual screening of the lower limb may need to be undertaken to assess the suitability of the intervention and to achieve optimal clinical outcomes.

## Conclusion

Associations between kinematic measures at the foot and moments at the knee indicate that increased rearfoot eversion, rearfoot internal rotation and forefoot inversion are associated with reduced KAM and KAAI during the stance phase of gait. These findings suggest that medial knee joint loading is reduced in people with OA who walk with greater foot pronation. Due to the high variability reported to load modifying interventions in people with medial compartment knee OA, individual screening of the lower limb may need to be performed to assess suitability for these interventions and to achieve optimal clinical outcomes.

## Abbreviations

OA: Osteoarthritis; KAM: Knee adduction moment; KAAI: Knee adduction angular impulse.

## Competing interests

HBM is Editor-in-Chief of the *Journal of Foot and Ankle Research*. It is journal policy that editors are removed from the peer review and editorial decision making processes for papers they have co-authored.

## Authors’ contributions

PL: designed and managed the study, collected and analysed the data drafted the manuscript. HBM: participated in the study design and assisted in the statistical analysis and data interpretation, helped to draft the manuscript. ADM: assisted in data collection, data analysis. JF, JB and NB have assisted in patient recruitment, grading x-ray severity and drafting the manuscript. PL, HBM and JF obtained the funding. All authors have read and approved the final version.
